# Chromosomal Polymorphism and Speciation: The Case of the Genus *Mazama* (Cetartiodactyla; Cervidae)

**DOI:** 10.3390/genes12020165

**Published:** 2021-01-26

**Authors:** David Javier Galindo, Gabriela Siqueira Martins, Miluse Vozdova, Halina Cernohorska, Svatava Kubickova, Agda Maria Bernegossi, Dita Kadlcikova, Jiri Rubes, José Maurício Barbanti Duarte

**Affiliations:** 1 Núcleo de Pesquisa e Conservação de Cervídeos, Faculdade de Ciências Agrárias e Veterinárias, Universidade Estadual Paulista—NUPECCE/FCAV/UNESP, 14884-900 Jaboticabal, Brazil; dgalindoh89@gmail.com (D.J.G.); agm.bernegossi@gmail.com (A.M.B.); 2 Laboratório de Dosagens Hormonais, Faculdade de Medicina Veterinária e Zootecnia, Universidade de São Paulo—FMVZ/USP, 05508-270 São Paulo, Brazil; gabriela.smartins88@gmail.com; 3 Department of Genetics and Reproductive Biotechnologies, Central European Institute of Technology—Veterinary Research Institute, 621-00 Brno, Czech Republic; vozdova@vri.cz (M.V.); cernohorska@vri.cz (H.C.); kubickova@vri.cz (S.K.); kadlcikova@vri.cz (D.K.); rubes@vri.cz (J.R.)

**Keywords:** cytogenetics, hybrids, post-zygotic barrier, sperm-FISH, Neotropical deer

## Abstract

Chromosomal polymorphism plays a major role in speciation processes in mammals with high rates of karyotypic evolution, as observed in the family Cervidae. One remarkable example is the genus *Mazama* that comprises wide inter- and intra-specific chromosomal variability. To evaluate the impact of chromosomal polymorphisms as reproductive barriers within the genus *Mazama*, inter-specific hybrids between *Mazama gouazoubira* and *Mazama nemorivaga* (MGO × MNE) and intra-specific hybrids between cytotypes of *Mazama americana* (MAM) differing by a tandem (TF) or centric fusion (Robertsonian translocations—RT) were evaluated. MGO × MNE hybrid fertility was evaluated by the seminal quality and testicular histology. MAM hybrids estimation of the meiotic segregation products was performed by sperm-FISH analysis. MGO × MNE hybrids analyses showed different degrees of fertility reduction, from severe subfertility to complete sterility. Regarding MAM, RT, and TF carriers showed a mean value for alternate segregation rate of 97.74%, and 67.23%, and adjacent segregation rate of 1.80%, and 29.07%, respectively. Our results suggested an efficient post-zygotic barrier represented by severe fertility reduction for MGO × MNE and MAM with heterozygous TF. Nevertheless, RT did not show a severe effect on the reproductive fitness in MAM. Our data support the validity of MGO and MNE as different species and reveals cryptic species within MAM.

## 1. Introduction

Chromosomal polymorphisms have played a meaningful role in speciation [1], by leading to the formation of efficient barriers to gene flow and subsequent differentiation process [2,3]. Among mammals, the family Cervidae stands out as one of the families with the highest presence of chromosomal polymorphisms, which is demonstrated in genera such as *Muntiacus,* whose diploid number ranges from 2n = 6/7 (*Muntiacus muntjak*) to 2n = 46 (*Muntiacus reevesi*) [4,5], and *Mazama*, whose diploid number ranges from 2n = 32–34 + Bs (*Mazama bororo*—MBO) to 2n = 70 + Bs (MGO) [6,7,8]. Intra-specific polymorphism is also present in several *Mazama* species (*Mazama nana*—MNA, 2n = 36–39 + Bs; MAM, 2n = 42–53 + Bs; MNE, 2n = 67–69 + Bs) [9,10,11] which, in part, justifies the great complexity in the species taxonomic definition and classification.

Regarding the genus *Mazama*, the occurrence of chromosomal rearrangements, mainly heterozygous RT, has been observed in MGO, the only holder of the ancestral karyotype within the genus [7,12,13]. Presence of these RTs denotes a high index of chromosomal fragility in this species, which has been previously tested and corroborated by doxorubicin-induced chromosomal aberrations [14,15]. Thus, we can hypothesize an ongoing speciation process in MGO [12]. Studies on deer mitochondrial DNA have suggest that MNE and MGO would not belong to the genus *Mazama*. Both of them standing in independent clades and distant from other *Mazama*, sharing the gray clade with other genera such as *Blastocerus*, *Ozotoceros*, and *Hippocamelus* [16,17]. In the meantime, this group is characterized by low levels of inter-specific chromosome difference and karyotypes with a high diploid number [11,16,17]. Due to their parapatric distribution [18] and morphological similarity, the differentiation between these two species has been the subject of extensive debate over the years, being demonstrated only recently through morphological [19,20], cytogenetic [11,20,21], and phylogenetic [20,22] analyzes.

Indeed, the comparison between MGO and MNE karyotypes have demonstrated that despite notable cytogenetic similarities, two chromosomal differences separate these species: (a) The presence of a MNE population with a rob(4;32), regarding to the base karyotype for the species, with a sex chromosome system XX/XY and a submetacentric X, different from the acrocentric X of MGO, and (b) The presence of an X-autosome TF in other MNE population, which resulted in a multiple sex chromosome system XX/XY1Y2 [7,8,11,20]. The occurrence of these rearrangements, by themselves, is already a strong indication of their possible role in the separation of these two species, although more evidence is needed to corroborate this statement [11,23].

Chromosomal polymorphisms are potent promoters of reproductive isolation since they can trigger a series of errors during meiosis in hybrids of different species or lineages, such as incorrect pairing of parental chromosomes, errors in chromosome segregation, and during crossing-over. These so-called meiosis defects have a deleterious effect on the individual’s reproductive fitness, leading to subfertility or sterility [1,24]. Although a description of morphophysiological evidence for reproductive isolation needs further investigation within the gray clade, this does not seem to be the case of the second clade of the genus *Mazama*, the red clade.

Regarding the red clade, what was traditionally reported as MAM today is considered a complex of cryptic species with two chromosomal lineages, one with high diploid number (Cytotypes Paraná—PR, 2n = 52/53, FN = 56; Santarém—SA, 2n = 50/51, FN = 56; Jarí—JA, 2n = 48/49, FN = 56; and Carajás—CA, 2n = 50/51, FN = 54) and one with a low diploid number (Cytotypes Juína—JU, 2n = 44/45, FN = 48; and Rondônia—RO, 2n = 42/43, FN = 46), all of them with wide geographical coherence [10]. Comparisons between cytotypes of the same lineage by G-banding showed minimal differences, such as TF or RT, from one cytotype to another [10].

A reproductive study on MAM showed that hybrids produced by crossbreeding of the two different chromosomal linages are sterile [25]. This indicated the occurrence of post-zygotic reproductive isolation between the MAM linages, which was associated with errors in meiotic recombination and gametic segregation due to several chromosomal differences, such as TF, RT, and inversions [25,26]. Hybrids between cytotypes of the same chromosomal lineage, with a chromosome number difference being equal to or less than 3 between the parents, were considered subfertile. Nonetheless, spermatogenesis was only evaluated in morphological and histological terms, without assessing the presence of chromosomally balanced or unbalanced gametes [25]. On the other hand, the presence of heterozygous RT in MAM probably only has a low effect on the reproductive fitness of the carrier [27].

This study aimed to assess the role of chromosomal polymorphism as a reproductive barrier and speciation mechanism within the genus *Mazama*. Thus, inter-specific hybrids between *M. gouazoubira* and *M. nemorivaga* (MGO × MNE) and intra-specific hybrids between *M. americana* (MAM) cytotypes differing by TF or RT were evaluated.

## 2. Materials and Methods

### 2.1. Species and Samples

Fibroblast tissue cultures prepared from skin biopsies according to standard protocols, testicular tissue, and sperm of *M. gouazoubira* (MGO), *M nemorivaga* (MNE), *M. americana* (MAM) cytotypes and hybrids, available at NUPECCE (Jaboticabal, São Paulo, Brazil), were used in the present study. For the inter-specific hybridization experiment, two hybrids between *M. gouazoubira* and *M. nemorivaga* and five pure bucks (*n* = 3, *M. gouazoubira* and *n* = 2, *M. nemorivaga*) were used and are described in Table 1. 

For the intra-specific hybridization experiment, two heterozygous Robertsonian translocation hybrids, three heterozygous TF hybrids, and two Carajás cytotype bucks from *M. americana* (MAM) were used. A detailed data of the animals is described in Table 2.

### 2.2. Whole-Chromosome Painting and Bacterial Artificial Chromosomes (BAC) Probes

Bovine whole-chromosome painting (WCP) probes were used for identification of chromosomes involved in the Robertsonian and Tandem fusions in animals analyzed in this study. Bovine whole chromosomes were isolated by flow sorting using MoFlo XDP Cell Sorter (Beckman Coulter, Brea, CA, USA) [29] or microdissected by PALM Microlaser system (Carl Zeiss MicroImaging GmbH, Munich, Germany) [30]. Once isolated, bovine chromosomes were used to produce WCP probes by DOP-PCR [31]. Probe labeling was performed during the secondary PCR with Green-dUTP or Orange-dUTP (Abbott Park, Chicago, IL, USA) [30].

For sperm-FISH, bovine BAC clones localized to the chromosomes involved in translocations were selected from the CHORI-240 cattle library (BACPAC Genomics, Emeryville, CA, USA). BAC DNA labeling was with digoxigenin-11-dUTP or biotin16-dUTP (Roche, Mannheim, Germany) was performed using BioPrime Array CGH Genomic Labeling Module (Invitrogen, Carlsbad, CA, USA). Detailed list of BACs used in the present study appears in Table S1.

### 2.3. FISH

FISH and sperm-FISH procedures were carried out as described in Vozdova et al. 2019 [32]. BAC probes labeled with digoxigenin-11-dUTP were detected with antidigoxigenin rhodamine (Roche Diagnostics, Indianapolis, IN, USA). BAC probes labeled with biotin-16-dUTP were detected with Avidin-FITC (Vector Laboratories, Inc., Burlingame, CA, USA). Hybridization signals were examined using Zeiss Axio Imager.Z2 fluorescence microscope (Carl Zeiss Microimaging GmbH, Jena Germany) equipped with appropriate fluorescent filters and the Metafer Slide Scanning System (MetaSystems, Altlussheim, Germany). Images of well-spread metaphase cells were captured and analyzed using ISIS3 software (MetaSystems, Altlussheim, Germany). 

### 2.4. Inter-Specific Hybrids (MGO × MNE) Reproductive Assessment

#### 2.4.1. Spermiogram

All animals went through at least one semen collection procedure once they achieved adulthood (<12 months of age). Electroejaculation procedure followed Favoretto et al. (2012) [33]. In short, all animals were anaesthetized intramuscularly with a combination of xylazine (1 mg/kg) and ketamine hydrochloride (7 mg/kg). Following sedation, a probe was inserted into the rectum and placed against the anterior wall close to the seminal vesicles. Each animal was submitted to sequential electroshocks increasing from 250 mA to 750 mA, with a mean duration of 3 s (and 3 s of rest). Three stimulation sequences of 10 shocks each were performed at intervals of 1–2 min [34]. Collected samples were maintained in microtubes (2 mL) at 37 °C in water bath until the beginning of analysis. Ejaculate color was determined by a single researcher to avoid any individual biases. Volume, total motility, sperm vigor, and sperm count were evaluated as described by Alvarez et al. (2020) [35]. Morphological analysis of the ejaculate was performed through the examination of wet preparations of fixed spermatozoa under phase contrast microscope. Morphological defects were classified according to their origin, to detect defects arising from an anomalous spermatogenesis (primary defects, resulting from testicular and secondary defects, resulting from inadequate maturation) [36].

#### 2.4.2. Testicular Histology

PG1, PG2, PN1, and H1 underwent unilateral orchiectomy after electroejaculation procedure. PG3, PN2, and H2 had their testicles collected immediately post-mortem. Testicular tissue was grossed into 1 cm thick sections, fixed in Bouin’s fixative for 24 h, processed for paraffin embedding, microtome-sectioned at 5-μm thickness, stained with hematoxylin and eosin, and imaged with a microscope. Then, 60 round or nearly round tubular profiles from each animal were randomly chosen and had diameter and epithelium height measured (Axio Vision v. 4.8.2, Carl Zeiss AG, Feldbach, Switzerland) size measurement tools were used). Ten sections of seminiferous tubules were analyzed to quantify the population of sperm cells. The results were presented with mean ± SD.

### 2.5. Intra-Specific Hybrids (MAM) Reproductive Assessment

#### 2.5.1. Semen Samples and Sperm Nuclei Preparation

Cryopreserved semen samples were obtained from NUPECCE’s germplasm bank. Ejaculates were collected and cryopreserved with Tris-egg yolk-glycerol extender [33]. To perform decondensation of the sperm nuclei, the method described by Rubes et al. (1999) was used, with slight modifications [37]. Briefly, semen samples were thawed at 37 °C for 20 s. Samples were transferred to a 2 mL Eppendorf tube, then washed with 500 µL of phosphate-buffered saline (PBS, pH 7.2), centrifuged at 380 g (5 min), and the supernatant discarded (repeated 3×). Pellet was resuspended in 500 µL of PBS containing 5 mM dithiothreitol (DTT), and incubated for 40–60 min, with slight homogenization every 10 min, then centrifuged. Pellet was washed in 300 µL of PBS (3×), and then fixed in Carnoy solution (3:1 methanol:acetic acid) (3×). Finally, samples were stored at −20 °C (30 min) in Carnoy solution. For dropping onto clean microscope slides, samples were diluted to a desired concentration.

#### 2.5.2. Sperm-FISH

The FISH protocol described in Section 2.3 was used with a slight modification for sperm denaturation. Briefly, spermatozoa were denatured in 1M NaOH for 6–10 min. BAC probes labeled with digoxigenin-11-dUTP and biotin-16-dUTP were detected with antidigoxigenin rhodamine and Avidin-FITC, respectively. Scoring of normal/balanced and unbalanced gametes was performed using Zeiss Axio Imager.Z2 fluorescence microscope. Only intact, non-overlapping gametes were scored using strict scoring criteria. The sperm was considered disomic if it showed two signals of the same color, size, and intensity, separated by a distance of at least one signal domain size. Diploid spermatozoa were differentiated from the double disomic ones by their larger size.

### 2.6. Statistical Analysis

Results for the histological measurements and the percentage of intratubular cells were presented by mean ± SD. All the results were submitted to Shapiro–Wilk normality test. Tubular diameter and germinal epithelium height did not present normal distribution therefore individual means were compared using non-parametric Kruskal-Wallis teste followed by pairwise Mann-Whitney U test with Bonferroni correction. Seminal parameters from hybrids between MGO and MNE and “pure” bucks were descriptively compared. Non-parametric Mann-Whitney exact test and Wilcoxon signed-rank test were used to compare frequencies of different segregation products between individuals and to compare FISH phenotypes per each chromosome, respectively. Meiotic segregation patterns were analyzed using the Kruskal-Wallis test and the difference between groups was obtained using the Dunn’s multiple comparison test, adjusted by Bonferroni. All analyzes were performed using Software R (R Foundation, 2020) [38] and *p* < 0.05 was considered significant.

## 3. Results

**Figure 1:**
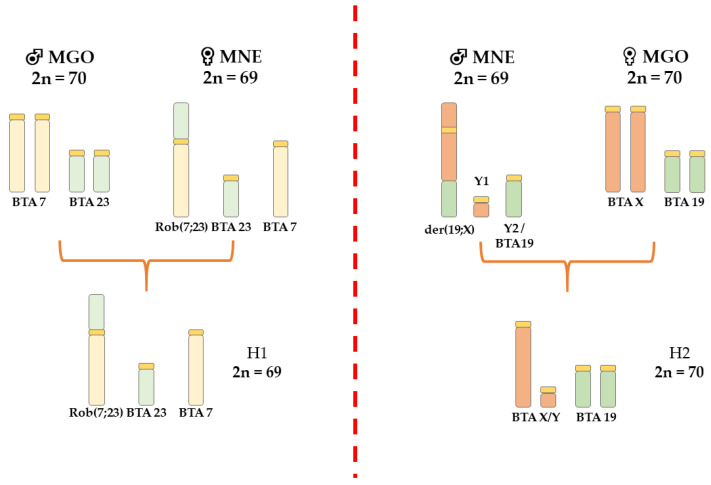
Schematic illustration showing the chromosomal polymorphism involved in the formation of MGO × MNE hybrids with orthologous bovine chromosomes.

**Figure 2:**
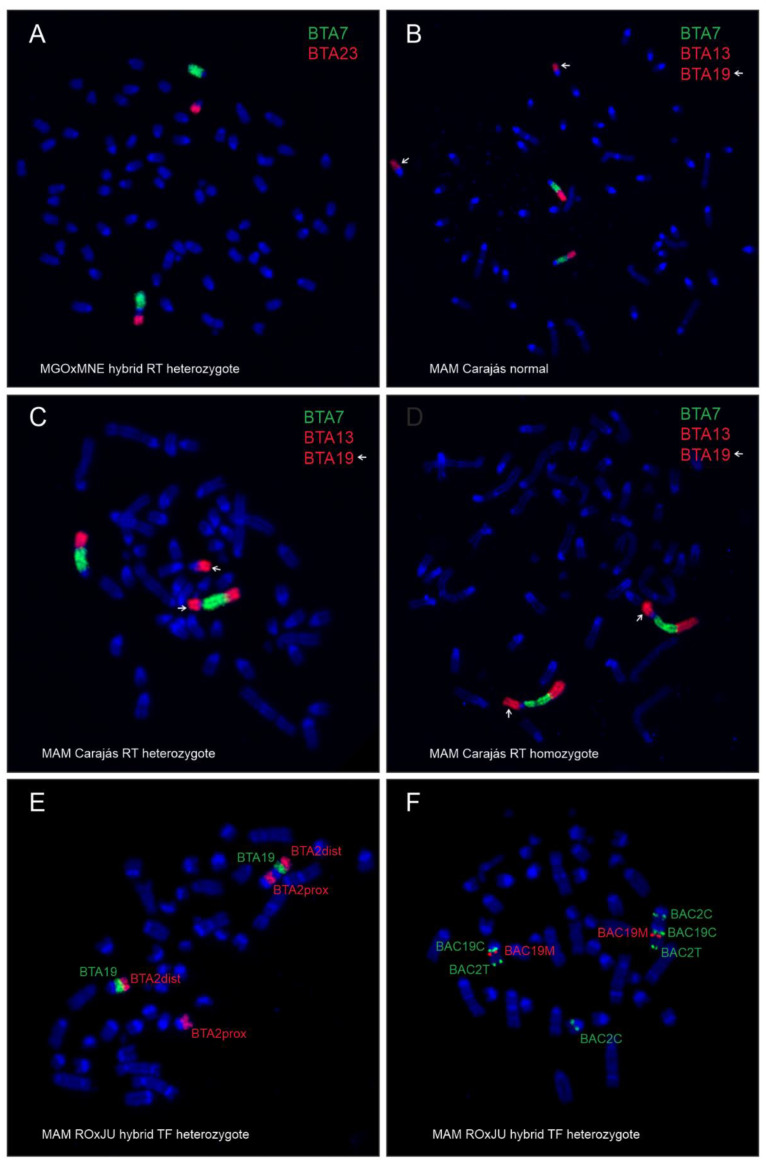
Metaphase chromosomes of the analyzed animals after FISH with bovine WCP (**A**–**E**) and BAC (**F**) probes. (**A**) Hybrid H1 (*M. gouazoubira* × *M. nemorivaga*) heterozygous for rob(4;32). (**B**) *M. americana* T297, Carajás cytotype, with normal karyotype. (**C**) *M. americana* T274, Carajás cytotype, heterozygous for rob(5;11). (**D**) *M. americana* T326, Carajás cytotype, homozygous for rob(5;11). (**E**) *M. americana* T343, Rondônia × Juína cytotype hybrid heterozygous for tandem fusion der(5;11) with WCP probes. (**F**) *M. americana*, Rondônia × Juína cytotype hybrid heterozygous for tandem fusion der(5;11) with BAC probes.

Using FISH with bovine WCP probes, we identified homologies between bovine chromosomes and the translocated chromosomes in the analyzed brocket deer hybrids. Chromosome differences between MGO and MNE identified by FISH with bovine WCP probes are shown in Figure 1. The FISH analysis of the hybrids showed that buck H1 obtained the rob(4;32) (Figure 2A) and the submetacentric X of MNE. The buck H2 did not inherit the X-autosomal fusion of MNE, but the acrocentric X of MGO.

Regarding MAM, differences between a non-translocated, heterozygous, and homozygous rob(5;11) in the Carajás cytotype are shown in Figure 2B–D. Difference between Rondônia (2n = 42/43) and Juína (2n = 44/45) cytotypes, as well as Carajás (2n = 50/51) and Paraná (2n = 52/53) cytotypes, was confirmed by FISH with bovine WCP and BAC probes, revealing a TF (centromere—telomere) (Figure 2E,F). Heterozygous TF in the hybrids was classified according to Abril (2009) [28], where a der(7;10) in Juína and a der(5;10) in Paraná are equivalent to the acrocentric chromosomes 4 in Rondônia and 3 in Carajás, respectively.

### 3.1. Inter-Specific Hybrids

**Figure 3:**
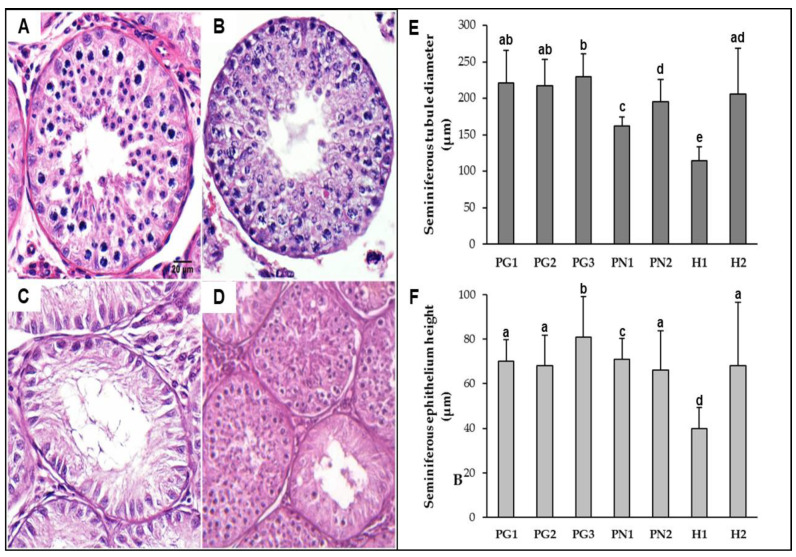
Morphometric differences between *M. gouazoubira* (PG), *M. nemorivaga* (PN) and inter-specific hybrids (H) testes (**A**–**D**); Histological sections of testes (scale bar = 20 mm); (**A**) Fertile PG buck; (**B**) fertile PN buck; (**C**) sterile hybrid buck (H1), note hypoplastic aspect of tubules and absence of spermatozoa in tubule lumen; (**D**) subfertile hybrid buck (H2), note juxtaposition of defective and functional seminiferous tubule cross-sections; (**E**) mean diameters of seminiferous tubules; and (**F**) mean seminiferous epithelium height. Columns followed by the same letter do not differ according to the Mann-Whitney U test (*p* < 0.05).

The fertility of pure and hybrids bucks (MGO × MNE) was assessed by testicular and sperm analysis. Photomicrographs of the testicular tissue revealed three distinct testis histology phenotypes (exemplified in Figure 3A–D) among pure animals and both hybrids. MGO and MNE testis (A,B) were considered totally functional, with multiple round tubules containing a plush spermatogenic epithelium. Morphometric measurements (Figure 3E,F) revealed larger tubules and thicker seminiferous epithelium, with PG3 presenting the highest mean values for tubular diameter and epithelium height among all individuals. Spermatogenesis was active and uniform in all sections analyzed, which was later confirmed by the quantification of sperm cells (Table 3) and the higher spermatid-to-spermatocyte ratio (SSR) values (1.50–4.19).

Regarding hybrids, histology phenotypes presented different levels of testicular hypoplasia as well as epithelial vacuolization suggestive of apoptosis. In H1, all the seminiferous tubules analyzed were hypoplastic (Figure 3C) with evidence of spermatogenesis interruption during the first meiosis (SSR = 0, Table 3). H1 showed significant lower mean diameter and epithelium height (*p* < 0.05) among all the animals analyzed. H2, in turn, seemed to be affected to a lesser extent, with the majority (90%) of seminiferous tubules being considered active (Figure 3D), even though spermatogenesis was complete in only part of them (demonstrated by reduction in later cell types, SSR = 0.84). Morphometric means for H2 did not significantly differ from most pure bucks (PG1, PG2, and PN2).

The seminal parameters are presented in Table 4. Overall, despite the species, pure individuals performed better than hybrids in most of the parameters evaluated. Seminal parameters of pure MGO and pure MNE remained rather consistent between both species and within the values of reference for them [39]. Regarding sperm morphology, in general, primary defects were more frequent in most animals. On the other hand, hybrid H1 was azoospermic, while the seminal analysis of hybrid H2 showed a remarkably low concentration, sparse motile sperm cells, and a high percentage of sperm defects (90%). Most of the defects were in the sperm flagellum and head.

### 3.2. Intra-Specific Hybrids

FISH with bovine BAC probes specific to the chromosomes involved in translocations was used to assess the fertility of heterozygous and homozygous translocation carriers in MAM. A total of 5000 and 2000 sperm nuclei were scored for RT and TF carriers, respectively. The sperm-FISH technique showed high specificity and sensitivity in red brocket deer sperm nuclei with bovine BAC probes hybridization rates higher than 99% in all cases. Results obtained for the RT and TF are presented in Table 5 and Table 6 and summarized below.

Regarding the RT carriers, the meiotic segregation patterns were not significantly different among the homozygous and heterozygous carriers and the control (Table 5). No significant differences were observed between the frequencies of nullisomies and disomies for any one of the analyzed chromosomes).

Regarding the heterozygous TF carriers, their segregation profiles (Table 6) were noticeably different when compared to RT cases described above. The MAM hybrids with heterozygous TFs showed lower rates of normal/balanced spermatozoa with a mean frequency of 67.23%, as well as higher rates of adjacent products with a mean frequency of 29.07%. Frequencies of nullisomies and disomies were not different for any one of the chromosomes. Hybrid T343 also shared the same RT presented for the Rondônia cytotype rob(7;20), inherited from its mother.

## 4. Discussion

Several studies on chromosomal polymorphism point out its key role in the formation of gene flow barriers between populations or species and, consequently, in the processes of adaptation and speciation [1,2,3]. In this context, the presence of chromosome heterozygosity is considered the main factor responsible for the formation of these barriers. Thus, a reduction in the reproductive fitness of the carriers might be caused by a hypothetical probability of errors in meiotic segregation and the formation of unbalanced gametes [1,2]. However, the real impact of these chromosomal rearrangements on the reproductive fitness of carriers and their subsequent impact within a population is not always fully understood. This knowledge gap worsens in wild species, where studies on the topic are scarce when compared to reports in domestic species [27,40,41,42,43,44,45].

Regarding the family Cervidae, the occurrence of chromosomal polymorphisms has been reported throughout the karyotype evolution of several species [7,8]. It is assumed that the ancestral karyotype of this family had 34 pairs of acrocentric autosomes, an acrocentric X, and a small submetacentric Y (2n = 70; FN = 70), given its presence in two species with long phylogenetic distance, such as *Hidropotes inermis* (Old world deer) and *M. gouazoubira* (New world deer) [5,7,8]. Thus, the karyotype evolution in the different genera of the family has been developed mainly by the reduction of the diploid number and the accumulation of chromosomal rearrangements such as inversions, RT or TF, as observed in the evolutionary history of the genus *Mazama* [7,8,9,10,11,46,47].

Hybridization evaluation between species or nearby lineages is one of the best approaches for those seeking to understand the diversification process [48]. In this study, we investigated the effect of chromosomal rearrangements on the fertility of hybrids between cytotypes of the same lineage (MAM) and between different species (MGO × MNE), to determine how these chromosomal polymorphisms could act as an effective barrier to genetic flow during parapatric or sympatric speciation in the genus *Mazama*.

### 4.1. Inter-Specific Hybrids

The sterility observed in hybrid animals is a way to irreversibly accelerate genetic divergences, preventing free gene flow between genetically different populations [49]. Traditionally, hybrid sterility is attributed to genetic incompatibilities between parental species, whether of chromosomal or genetic origin [50]. Although in most animals, incompatibilities mediated by deleterious interactions between genes are considered the primary cause of hybrid inaptitude (Dobzhansky-Müller model). The results of cytogenetic analyzes of the MGO × MNE hybrids most likely indicate that the occurrence of post-zygotic reproductive isolation between MGO and MNE is probably linked to numerical and structural chromosomal differences. These differences lead to the accumulation of heterozygous chromosomal rearrangements in the hybrids and may trigger anomalous pairing during meiosis, resulting in gametogenesis failures and unbalanced gamete production [51].

Even though inter-individual variation among animals was evident, in general, all seminal, morphological, and most histological reproductive parameters observed in pure animals (PG1, PG2, PG3, PN1, and PN2) were superior to those obtained for hybrids, being within expected for their respective species [39,52]. In contrast, evidence of fertility reduction varied between the hybrids, showing different effects of chromosomal differences found between the parent’s karyotypes.

The effect of the chromosomal rearrangements accumulation on hybrid reproductive fitness was especially evident in H1, with a rob(4;32) and a submetacentric X inherited from the mother (MNE). In its spermiogram, this animal demonstrated complete interruption of spermatogenesis, which was reflected in azoospermia. In this case, presumed sterility could be attributed to multiple chromosomal pairing failures during meiosis, getting worse when differences between parent karyotypes are greater [53]. Thus, the H1 karyotype (2n = 69 + 0–3 Bs) was the most discrepant concerning the pattern of parental species among the analyzed MGO × MNE hybrids.

In most cases of hybrid sterility, associations between cell death and meiosis occur between pachytene and spermiogenesis, which results in high attrition rates in the pachytene of meiosis I [54]. Similar patterns in the histological analysis of H1 cell types suggested spermatogenic interruption. Moreover, the total hypoplasia of seminiferous tubules observed in H1, frequently described in infertile hybrids [55,56,57], is a direct consequence of the spermatogenesis interruption during meiosis I. The absence of differentiated germ cells results in a decrease in tubular diameter and height of seminiferous epithelium, aspects that in H1, obtained the lowest averages among all animals analyzed. Similar conformations have been reported in other hybrid forms such as donkeys [58], rats [57], and within the MAM cytotype complex itself [25].

Despite having obtained better performance than H1 in all reproductive analyzes, mostly functional tubular structure, and no chromosomal translocation, the fertility of the H2 hybrid was also severely affected by chromosomal differences. The severe subfertility showed by H2 reinforces the importance of the role of chromosomes in the process of reproductive isolation, even when the rearrangements are not so apparent. Seminal analysis of this animal revealed an ejaculate with extremely low volume and concentration, irrelevant motility, and a high prevalence of sperm defects. This low seminal quality is the result of a series of structural, pathological, and functional changes at the testicular level: H2 showed hypoplasia in part of its seminiferous tubules and the presence of cells with a pycnotic nucleus and epithelial vacuolization, suggestive of the occurrence of apoptosis in both functional and hypoplastic tubules. Moreover, H2 also showed a low conversion rate between spermatids and spermatocytes (SRR = 0.84) when compared with pure animals. All of this evidence points to the loss of germinal epithelium and cell degeneration, typically found in hybrid forms [57,59].

Finally, it is worth remembering that although the presence of sperm in a hybrid ejaculate has been described in several inter-specific crossbreeding [55,59,60,61,62], it does not guarantee its fertility. Chromosomal non-disjunction during anaphase I is the second leading cause of reduced fertility in these animals since heterozygous configurations of hybrids undergo an anomalous separation process leading to the formation of unbalanced gametes (aneuploidy) and non-viable embryos [59]. Thus, it is likely that, similar to what was observed in intra-specific MAM hybrids in this study, future FISH analysis of H2 also reveals a high rate of unbalanced gametes.

Since the pre-zygotic reproductive barrier between MNE and MGO is fragile [63], the post-zygotic barrier for sterility of the hybrid seems to keep these two species isolated and evolving independently. Even with wide geographical contact between the Amazon (MNE habitat) and the Cerrado (MGO habitat) for more than 2000 km.

### 4.2. Intra-Specific Hybrids

A previous study carried out in MAM, demonstrated that hybrids with the presence of heterozygous TF presented seminal parameters similar to those presented by pure animals of the different lineages (volume: 270 µL vs. 135 µL; motility: 75% vs. 77.5%; concentration: 2.22 sptz × 10^9^/mL vs. 3.81 sptz × 10^9^/mL; and pathologies: 47.25% vs. 30%, for heterozygous TF hybrids and pure animals, respectively) [25]. Thus, the fertility of the hybrids could not be defined or ruled out, which is why they were considered subfertile. Because of this, we decided to perform the technique of sperm-FISH to estimate the proportion of normal/balanced and unbalanced spermatozoa in bucks with heterozygous rearrangements and animals from crossbreeding between cytotypes of the same lineage in MAM. The proportion of meiotic products from adjacent segregation modes in RT carriers analyzed in this study is consistent with reports for domestic species such as bulls, boars and mice (2.58–5.42%, 3.16%, and 8–11.5%, respectively) [64,65,66,67]. These findings may suggest a low negative effect on the reproductive fitness of heterozygous carriers of RT reported here for MAM, unlike that reported for several RT in humans where there is a wide variation in reproductive impact (0.2–49.1% of adjacent segregation products) [68].

Our results are in agreement with a previous report focused on the synaptonemal complex analysis of the same heterozygous rob(7;20) carrier (T269) [27], where results suggested a highly unlikely formation of unbalanced gametes for this RT. Similar results for synaptonemal complex analysis focused on the effect of centromeric fusion on meiosis and reproduction of cattle, goitered gazelle, and impala have been reported [40,69,70]. In fact, the NUPECCE’s breeding records indicate that this rob(7;20) carrier was used for breeding purposes and produced 4 fawns, not exhibiting any obvious reproductive impairment. Regarding the rob(5;11), the heterozygous carrier produced a non-translocated female fawn, also suggesting no reproductive impairment. On the other hand, no reproductive records were available for the homozygous carrier. However, our findings suggest that the homozygous translocation could offer greater stability during the meiotic segregation, not affecting its reproductive fitness, and showing a meiotic segregation pattern similar to the control values. The presence of homozygous translocation suggests a possible fixation of this chromosomal polymorphism in free-living populations, opening the possibility of future speciation processes. However, our results on the meiotic segregation patterns of carriers, both homozygous and heterozygous rob(5;11), would suggest an apparent gene flow between these populations. Thus, every single RT must be assessed to understand its potential effect on the reproductive fitness of the carriers. Errors in meiosis are the result of the behavior of those chromosomes involved in the translocation and their trivalent during the first meiotic segregation. [40,68].

In this study, we also analyzed three heterozygous TF carriers produced in captivity between *Mazama americana* cytotypes of the same chromosomal lineage (*n* = 2, Rondônia × Juína cytotypes; *n* = 1, Carajás × Paraná cytotypes) [10]. Although TF are chromosomal rearrangements present in the evolutionary history of cervids, they have been previously related with reduction in fertility in animal [71,72,73]. A previous study reported subfertile male hybrids from MAM cytotypes of the same chromosomal lineage and azoospermic hybrids from different chromosomal lineages [25]. Azoospermia was attributed to the great karyotypic differences, a meiotic arrest in spermatocyte stage, and errors in meiotic segregation for hybrids between different lineages, providing an adequate post-zygotic reproductive barrier and suggesting the presence of different species [25]. In this study, MAM hybrids heterozygous for TF showed the highest rate of unbalanced spermatozoa of all analyzed *Mazama* males. This can explain the previously reported subfertility of Rondônia × Juína hybrids, carrying a heterozygous TF, which did not show any significant compromise in seminal quality or testicular histology [25].

Also, it is important to mention that hybrid T343 also carried a heterozygous rob(7;20), which might have increased the errors in meiotic chromosome pairing, leading to a greater error in meiotic segregation in this buck. Thus, the red brocket male T269 only heterozygous for the rob(7;20), or hybrid T347 only heterozygous for the der(7;10), showed unbalanced spermatozoa rates of 2.20 and 30.50%, respectively. Regarding hybrids T347 and T421, our data presented about 70% balanced gametes suggesting a subfertility status, similar to Salviano et al. (2017) and contrasting the estimates of 50% aneuploid gametes made by White et al. (1967) [74] for heterozygous TF. However, if we consider a hypothetical 1:1 ratio between gametes carrying or not the TF, we would have a frequency of 35% for each phenotype. This will be, only a 35% chance of successful reproduction in a backcross of the T347 hybrid with a female of cytotype Rondônia or Juína, and the T421 hybrid with a female of cytotype Carajás or Paraná, suggesting virtual sterility of the hybrids similar to the *Otomys irroratus* case [73], and dismissing the previous description of subfertility for hybrids carrying heterozygous TF in MAM, made by Salviano et al. (2017) [25].

We report the first production of hybrids between MGO and MNE, which were viable until maturity, but presumably infertile. There are no reports of hybrids in the wild, although a weak pre-mating isolation barrier between species has been observed in captivity [63]. Regarding MAM, reports of captive crossbreeding between cytotypes already exist [25,75], which are explained by the verified lack of a clear pre-mating barrier [26]. However, it is difficult to say that this can happen in nature, despite the geographical proximity between MGO and MNE, as well as between the MAM cytotypes. Therefore, there is a clear need for a better understanding of chromosomal polymorphisms between species and intra-specific populations to elucidate their role in forming barriers to gene flow within the genus *Mazama*, the isolation from former populations, and subsequent adaptation/speciation. Moreover, meiotic segregation assessment in hybrids and carriers of heterozygous chromosomal translocations is presented as a mandatory tool for estimating the impact of chromosomal polymorphisms in both the reproductive fitness of carriers and in *Mazama* speciation processes. Thus, leaving the morphological evaluation of the gametes as a complementary assessment.

## Figures and Tables

**Table 1:** Summary of chromosomal data from *M. gouazoubira*, *M. nemorivaga*, and inter-specific hybrids.

Animal	Species	2n	FN	Translocations		B
				RT	Multiple Sexual System	
PG1	M. gouazoubira	70	70	-	No	0–2
PG2	M. gouazoubira	70	70	-	No	0–2
PG3	M. gouazoubira	70	70	-	No	0–2
PN1	M. nemorivaga	68	72	rob(4;32)(4;32) ^a^	No	1–9
PN2	M. nemorivaga	67	70	rob(4;32)(4;32) ^a^	Yes ^a^	2–5
H1	MGO 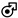 × MNE 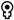	69	72	rob(4;32) ^a^	No	0–3
H2	MNE 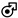 × MGO 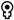	70	70	-	No	0–2

2n = chromosome number; FN = fundamental number; RT = Robertsonian translocation; TF = tandem fusion; B = supernumerary chromosomes. ^a^ Chromosome classification according to standard karyotype for *M. nemorivaga* [11].

**Table 2:** Summary of chromosomal data from *M. americana* carriers of chromosomal translocations, hybrids of different MAM cytotypes, and non-translocated animals.

Animal	Cytotypes	2n	FN	Translocations		B
				RT	TF	
T297 ^a^	Carajás	51	54	-	-	2–3
T274	Carajás	50	54	rob(5;11) ^b^	-	3
T326	Carajás	49	54	rob(5;11)(5;11) ^b^	-	3–4
T269	Rondônia	42	46	rob(7;20) ^c^	-	3–5
T343	Juína 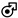 × Rondônia 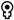	43	47	rob(7;20) ^c^	der(7;10) ^d^	2–3
T347	Rondônia 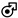 × Juína 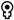	44	47	-	der(7;10) ^d^	2–4
T421	Paraná 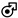 × Carajás 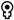	52	55	-	der(5;10) ^e^	-

2n = chromosome number; FN = fundamental number; RT = Robertsonian translocation; TF = tandem fusion; B = supernumerary chromosomes. ^a^ Control bucks, normal karyotype. ^b^ Chromosome classification according to Carajás cytotype [10]. ^c^ Chromosome classification according to Rondônia cytotype [10,27]. ^d^ Chromosome classification according to Juína cytotype [28]. Rondônia chromosome 4 = tandem fusion of Juína chromosomes 7 + 10. ^e^ Chromosome classification according to Paraná cytotype [28]. Carajás chromosome 3 = tandem fusion of Paraná chromosomes 5 + 10.

**Table 3:** Mean ± standard deviation of the percentages of cell types of the seminiferous epithelium of adult males *M. gouazoubira* (PG), *M. nemorivaga* (PN) and inter-specific hybrids (H).

Animal	Spermatogonia A (%)	Spermatogonia B (%)	Leptotenes/Zygotenes (%)	Pachytenes (%)	Round Spermatids (%)	Sertoli Cells (%)	SSR ^a^
PG1	17.36 ± 12.70	22.63 ± 10.49	9.61 ± 4.30	10.53 ± 2.75	21.45 ± 12.34	18.42 ± 2.91	2.03
PG2	6.34 ± 2.20	12.67 ± 7.08	21.42 ± 22.00	16.16 ± 5.81	33.40 ± 15.23	10.01 ± 2.46	2.06
PG3	11.03 ± 7.98	12.76 ± 7.44	16.77 ± 31.65	14.54 ± 14.46	33.36 ± 23.08	11.53 ± 4.00	2.29
PN1	15.61 ± 4.69	13.33 ± 6.81	22.28 ± 10.90	15.70 ± 4.43	23.86 ± 14.72	9.22 ± 1.43	1.50
PN2	9.42 ± 7.04	8.22 ± 2.45	16.38 ± 13.49	10.47 ± 5.72	43.95 ± 20.33	11.56 ± 4.12	4.19
H1	41.64 ± 4.55	29.79 ± 2.39	9.42 ± 0.00	0.00	0.00	19.15 ± 3.65	0.00
H2	15.52 ± 3.39	15.64 ± 6.82	22.79 ± 8.36	18.18 ± 5.23	15.27 ± 6.17	12.60 ± 2.01	0,84

^a^ Spermatid-to-spermatocyte ratio.

**Table 4:** Seminal parameters of adult bucks of *M. gouazoubira*, *M. nemorivaga*, and inter-specific hybrids.

Animal	Volume (μL)	Concentration (10^9^/mL)	Color	Motility (%)	Vigor (0–5)	Defects (%)		Normal Sperm (%)
						Primary	Secondary	
PG1	375	0.57	White	40	2	37.0	5.0	58.0
PG2	240	3.34	White	65	4	29.5	13.5	57.0
PG3	270	2.32	White	60	3	13.5	13.0	73.0
PN1	375	2.25	Reddish ^a^	70	3	35.0	13.5	51.5
PN2	60	2.71	Reddish ^a^	90	4	4.0	39.0	57.0
H1 ^b^	160	-	Clear	-	-	-	-	-
H2	50	0.02	Watery	<1	0	66.0	24.0	10.0

^a^ Considered physiologically normal for the species [39]. ^b^ Azoospermic.

**Table 5:** Sperm meiotic segregation in Robertsonian translocation carriers of *Mazama americana*, including a non-translocated buck as control.

	Robertsonian Translocation (A;B) (%)							
FISH Phenotype	T269 rob(7;20) ^a,d^		T274 rob(5;11) ^a,e^		T326 rob(5;11) ^b,e^		T297 ^c,e^	
**Normal/Balanced**	4890	(97.80)	4884	(97.68)	4949	(98.98)	4926	(98.52)
Nullisomy A	36	(0.72)	21	(0.42)	18	(0.36)	19	(0.38)
Disomy A	7	(0.14)	10	(0.20)	6	(0.12)	3	(0.06)
Nullisomy B	25	(0.50)	33	(0.66)	8	(0.16)	17	(0.34)
Disomy B	29	(0.58)	19	(0.38)	7	(0.14)	7	(0.14)
**Total adjacent**	97	(1.94)	83	(1.66)	39	(0.78)	46	(0.92)
Disomy A + B	1	(0.02)	9	(0.18)	0	(0.00)	7	(0.14)
Nullisomy A + B	1	(0.02)	9	(0.18)	6	(0.12)	2	(0.04)
Diploidy	7	(0.14)	10	(0.20)	5	(0.10)	19	(0.38)
Others ^f^	4	(0.08)	5	(0.10)	1	(0.02)	0	(0.00)
**Total unbalanced**	110	(2.20)	116	(2.32)	51	(1.02)	74	(1.48)
**TOTAL**	5000	(100)	5000	(100)	5000	(100)	5000	(100)

^a^ Heterozygous carrier. ^b^ Homozygous carrier. ^c^ Non-translocated buck (normal karyotype). ^d^ Buck analyzed with bovine BAC probes 17C and BAC 25M. ^e^ Buck analyzed with bovine BAC probes 13T and 19T. ^f^ Other, less frequent signal combinations.

**Table 6:** Sperm meiotic segregation in heterozygous tandem fusion carrier hybrids of *Mazama americana* cytotypes, including a non-translocated buck as control.

	Tandem Fusion (A;B) (%)							
FISH Phenotype	T343 der(7;10) ^a^		T347 der(7;10) ^a^		T421 der(5;10) ^b^		T297 ^b,c^	
**Normal/Balanced**	1139	(56.95)	1390	(69.50)	1505	(75.25)	1970	(98.50)
Nullisomy A	328	(16.40)	203	(10.15)	166	(8.30)	6	(0.30)
Disomy A	249	(12.45)	170	(8.50)	144	(7.20)	5	(0.25)
Nullisomy B	124	(6.20)	109	(5.45)	56	(2.80)	8	(0.40)
Disomy B	82	(4.10)	67	(3.35)	46	(2.30)	4	(0.20)
**Total adjacent**	783	(39.15)	549	(27.45)	412	(20.60)	23	(1.15)
Disomy A + B	18	(0.90)	13	(0.65)	26	(1.30)	4	(0.20)
Nullisomy A + B	11	(0.55)	12	(0.60)	11	(0.55)	3	(0.15)
Diploidy	15	(0.75)	11	(0.55)	12	(0.60)	0	(0.00)
Others ^d^	34	(1.70)	25	(1.25)	34	(1.70)	0	(0.00)
**Total unbalanced**	861	(43.05)	610	(30.50)	495	(24.75)	30	(1.50)
**TOTAL**	2000	(100)	2000	(100)	2000	(100)	2000	(100)

^a^ Buck analyzed with bovine BAC probes 2P and BAC 19T. ^b^ Buck analyzed with bovine BAC probes 3T and 28M. ^c^ Non-translocated buck (normal karyotype). ^d^ Other, less frequent signal combinations.

## Data Availability

The data presented in this study are available in the article and supplementary material.

## Supplementary Materials

The following are available online at https://www.mdpi.com/2073-4425/12/2/165/s1, Table S1: List of bovine BAC clones used in the present study for detection of bovine (*Bos Taurus*—BTA) homologies with brocket deer chromosomes involved in translocations and for sperm-FISH.
