# Supplementary material for: Chromosomal Polymorphism and Speciation: The Case of the Genus Mazama (Cetartiodactyla; Cervidae)
Source: Genes (Basel). 2021 Jan 26;12(2):165. doi: 10.3390/genes12020165 (PMC7911811; doi:10.3390/genes12020165)
Supplement: Supplementary file 1 [file genes-12-00165-s001.zip › Supplementary Materials.docx]

**Table S1.** List of bovine BAC clones used in the present study for detection of bovine (*Bos taurus* – BTA) homologies with brocket deer chromosomes involved in translocations and for sperm-FISH.

| Chromosome region | | Cattle location (Mb) | BAC clone (probe name) |
| --- | --- | --- | --- |
| Cattle | **Mazama** |  |  |
| BTA 2 | MAM-JU 10 | 9.510 – 9.718 | CH240-42D15 (BAC 2C) |
|  |  | 91.323 – 91.543 | CH240-186F21 (BAC 2P) |
|  |  | 135.511 – 135.718 | CH240-437C7 (BAC 2T) |
| BTA 3 | MAM-PA 5 | 120.614 – 120.777 | CH240-250C11 (BAC 3T) |
|  |  | 120,807 – 120.9.2 | CH240-106D7 (BAC 3T) |
| BTA 13 | MAM-CA 5 | 74.679 – 74.851 | CH240-332D20 (BAC 13T) |
|  |  | 75.027 – 75.262 | CH240-278J11 (BAC 13T) |
| BTA 17 | MAM-RO 7 | 3.909 – 4.129 | CH240-63H8 (BAC 7C) |
|  |  | 4.140 – 4.334 | CH240-175F4 (BAC 7C) |
| BTA 19 | MAM-JU 7  MAM-CA 11 | 9.123 – 9.343 | CH240-130E10 (BAC 19C) |
|  |  | 34.353 – 34.575 | CH240-50L8 (BAC 19M) |
|  |  | 55.619 – 55.852 | CH240-106P5 (BAC 19T) |
|  |  | 55.911 – 56.097 | CH240-188A20 (BAC 19T) |
| BTA 25 | MAM-RO 20 | 21.271 – 21.511 | CH240-89A17 (BAC 25M) |
| BTA 28 | MAM-PA 10 | 24.741 – 24.943 | CH240-108O21 (BAC 28M) |
| BTA = *Bos taurus*; MAM = *Mazama americana*; JU = Juína cytotype; PA = Paraná cytotype; CA = Carajás cytotype; RO = Rondônia cytotype. | | | |
